# E-cigarette use among adolescents: Are we doing enough?

**DOI:** 10.1016/j.eclinm.2022.101623

**Published:** 2022-08-17

**Authors:** eClinicalMedicine

There have been growing concerns that the use of electronic cigarettes (e-cigarettes), commonly known as vaping, is becoming increasingly popular among adolescents, particularly in Europe. Substantiating these concerns, the 2022 Action on Smoking and Health Survey of 11–18-year-olds in Great Britain showed that trends in the use of e-cigarettes in this age group have risen sharply in the past 12 months, with the proportion of 11–15-year-olds and 16–17-year-olds currently using e-cigarettes in 2022 almost or more than doubling since 2021 to 4·0% and 14·1%, respectively. E-cigarette use in the WHO European region also poses a concern, with reports of children as young as 11 years having tried e-cigarettes at least once in 17 European study sites. Furthermore, the age-adjusted prevalence of e-cigarette use among 11-17-year-olds has more than doubled in Georgia and Italy, and almost doubled in Latvia since 2014. Despite a decline in the use of e-cigarettes among adolescents in the USA between 2019 and 2021, more than 2 million middle school and high school students reported being current users of e-cigarettes in 2021. Data on adolescent e-cigarette use in Asian countries, South America, and low-income countries such as India and Africa are scarce, but available data suggest that the prevalence might be lower in some Asian countries (eg, 3·5% in China) and Brazil (2·1%), than in Europe or the USA. Although most adolescents have never tried e-cigarettes, and there have been promising reductions in underage combustible cigarette smoking, the recent rise in the use of e-cigarettes among youths in some countries has sparked health warnings from respiratory health experts.

E-cigarettes are battery-powered devices, in which a solution containing water and chemicals, with or without nicotine or flavourings, is heated electronically to produce an aerosol that is inhaled by the user. The solution can also contain a cocktail of potentially harmful chemicals, such as ultrafine particles, volatile organic compounds, heavy metals, and diacetyl—a compound in flavourings that has been linked to lung disease. The landscape of tobacco products is evolving and expanding, but a wide range of e-cigarette devices of varying shapes and sizes are available. Most e-cigarettes contain nicotine at concentrations ranging from 3 mg/mL to 36 mg/mL; a single pod can contain up to the same amount of nicotine as a pack of 20 regular cigarettes.

E-cigarettes are considered less harmful than combustible cigarettes, and although the evidence is not definitive, they are endorsed by public health bodies as an effective tool to quit smoking. However, data on the safety and the long-term health effects of e-cigarettes on human health are by no means clear. Evidence suggests that vaping can reduce lung function, increase the risk of respiratory infections, and exacerbate existing lung conditions, such as asthma or bronchitis. Unsurprisingly, data on the short-term and long-term effects of e-cigarette use and nicotine exposure in adolescents are also scarce. Worryingly, e-cigarette use has been associated with chronic bronchitis symptoms in adolescence. Research also suggests that nicotine can negatively affect adolescent brain development, potentially leading to changes in mood, learning, attention, and impulse control. Nicotine addiction can also be a source of stress, and nicotine withdrawal symptoms can have a substantial effect on mood and behaviour. A bi-directional association between e-cigarette and combustible cigarette use and depressive symptoms among adolescents has also been identified. Of particular concern, e-cigarette use in adolescents, particularly among those who have never smoked, could even be a gateway to smoking.

Given the potential detrimental effects of e-cigarettes on adolescent health and development, it is important to understand why so many youths are vaping. According to the 2021 US Annual National Youth Tobacco Survey, the most common reason for first trying e-cigarettes was because a friend used them (57·8%), and the most common reason for current use was due to feelings of stress, anxiousness, or depression (43·4%), closely followed by the “high or buzz” from nicotine (42·8%). Most (84·7%) current e-cigarette users reported using a flavoured device, with fruit (71·6%) and candy, desserts, or other sweets (34·1%) flavours being the most popular. Similarly, the aforementioned UK Action on Smoking and Health survey results indicated that the most common reason for 11-17-year-old smokers to use e-cigarettes was because they liked the flavours (20·8%). Despite being illegal to sell e-cigarettes to those under the age of 18 years in the UK, shops were the most common source of e-cigarettes (47%), and over half (56%) of youths reported being aware of e-cigarette promotion, most frequently in shops or online (mostly on TikTok and Instagram). Interestingly, when given a choice between branded packs with or without brand imagery, significantly fewer youths expressed an interest in those without brand imagery, irrespective of whether they used e-cigarettes.

From these data, it is clear that the use of flavours, promotion, branding, lack of age verification by e-cigarette vendors, and peer pressure are key drivers for e-cigarette use among youths. Although regulation of e-cigarettes varies worldwide, many countries have implemented legislation that targets some of these and other factors already, while others simply ban e-cigarettes entirely. Unlike the USA and countries in the WHO European region, which have either banned or only permit certain flavours, or mandated plain packaging or large graphic warnings, there is limited regulation on the packaging and sale of flavoured e-cigarettes in the UK. One promising move in Europe is the recent (June, 2022) proposal by the European Commission to ban the sale of flavoured heated tobacco products in the EU, as part of their beating cancer plan.

E-cigarettes contain a highly addictive substance and potentially harmful chemicals, and they should not be accessible to adolescents. It is clear that tight regulation of the sale, promotion, and content of e-cigarettes is needed to prevent the initiation and use, and increase the safety of these products. Sadly, the available evidence suggests that e-cigarettes could halt or even reverse the declining use of tobacco products among adolescents. Moving forward, it is important that high quality data on the health effects of e-cigarettes, as well as on effective vaping prevention messaging to adolescents, are collected to inform targeted national and global e-cigarette control and prevention strategies.

